# The Unique Properties of Placental Mesenchymal Stromal Cells: A Novel Source of Therapy for Congenital and Acquired Spinal Cord Injury

**DOI:** 10.3390/cells10112837

**Published:** 2021-10-22

**Authors:** Edwin S Kulubya, Kaitlin Clark, Dake Hao, Sabrina Lazar, Arash Ghaffari-Rafi, Tejas Karnati, Julius Okudu Ebinu, Marike Zwienenberg, Diana L Farmer, Aijun Wang

**Affiliations:** 1 Department of Surgery, Davis School of Medicine, University of California, Sacramento, CA 95817, USA; ekulubya@ucdavis.edu (E.S.K.); kcclark@ucdavis.edu (K.C.); dkhao@ucdavis.edu (D.H.); lazars@amc.edu (S.L.); Dlfarmer@ucdavis.edu (D.L.F.); 2 Department of Neurological Surgery, Davis School of Medicine, University of California, Sacramento, CA 95817, USA; aghaffarirafi@ucdavis.edu (A.G.-R.); tkarnati@ucdavis.edu (T.K.); joebinu@ucdavis.edu (J.O.E.); mzwien@ucdavis.edu (M.Z.); 3 Institute for Pediatric Regenerative Medicine, Shriners Hospitals for Children, Sacramento, CA 95817, USA; 4 Department of Biomedical Engineering, UC Davis, Davis, CA 95616, USA

**Keywords:** mesenchymal stromal cells, extracellular vesicles, spinal cord injury, spina bifida, myelomeningocele

## Abstract

Spinal cord injury (SCI) is a devasting condition with no reliable treatment. Spina bifida is the most common cause of congenital SCI. Cell-based therapies using mesenchymal stem/stromal cells (MSCS) have been largely utilized in SCI. Several clinical trials for acquired SCI use adult tissue-derived MSC sources, including bone-marrow, adipose, and umbilical cord tissues. The first stem/stromal cell clinical trial for spina bifida is currently underway (NCT04652908). The trial uses early gestational placental-derived mesenchymal stem/stromal cells (PMSCs) during the fetal repair of myelomeningocele. PMSCs have been shown to exhibit unique neuroprotective, angiogenic, and antioxidant properties, all which are promising applications for SCI. This review will summarize the unique properties and current applications of PMSCs and discuss their therapeutic role for acquired SCI.

## 1. Introduction

Cell-based therapies for spinal cord injury (SCI) have been investigated in preclinical and clinical trials with limited success [1,2]. The majority of these investigations use mesenchymal stem/stromal cells (MSCs) derived from adult tissue, including bone marrow, adipose, and umbilical cord tissues [2]. Recently, placental early gestational chorionic villus-derived MSCs (PMSCs) have been applied to treat spina bifida in utero [3].

Spina bifida, a congenital form of SCI, results from the incomplete closure of the spinal canal during development. Due to this incomplete closure, the fetal spinal cord is subjected to physical and chemical trauma in utero, causing prenatal damage to the exposed spinal cord [4,5]. Children born with spina bifida are often paralyzed in their lower extremities and suffer from bowel and bladder incontinence [4]. Studying this congenital form of SCI could provide useful insights into developing an effective cellular therapy for acquired and adult SCI.

Placental tissues have been investigated for their potential for autologous treatment in fetal surgery. Fetal surgery for spina bifida showed improved motor outcomes in the landmark Management of Myelomeningocele Study (MOMS) [6]. Despite this success, there were still patients who could not walk or had bowel and bladder incontinence. Novel cellular therapy has the potential to improve those outcomes. The placenta, which maintains fetal immunological tolerance, is known to contain unique MSCSs which display immunomodulatory, neuroprotective, angiogenic, and antioxidant properties [7]. The current review will explore the unique properties and current applications of PMSCs and their potential for both congenital and acquired SCI treatment.

## 2. Spinal Cord Injury Pathogenesis

Prior to discussion of treatment for congenital and acquired SCI, a robust understanding of the pathogenesis underlying SCI is required. Historically, SCI has been stratified into two phases: primary and secondary injury [8,9]. Primary injury involves the immediate period (i.e., within 2 h) after direct mechanical forces are imparted onto the spinal cord [8,9]. Subsequently, secondary injury ensues, a chronic and progressive phase involving vasogenic and cytotoxic edema, free radical production, neurotransmitter-mediated excitotoxicity, vascular dysfunction (i.e., hemorrhage, ischemia, necrosis), oligodendrocyte death (demyelination), reactive astrocytosis, postponed apoptotic cell death, and protracted Wallerian degeneration extending past six months from injury [8,9,10].

Primary injury, which encompasses the first two hours after SCI, sets the precedent for SCI severity and outcome [8,9,10,11,12]. The initial mechanical force on the cord—including but not limited to shearing, laceration, compression, or sudden impact—directly damages neural substrate and blood vessels, triggering the cascade of spinal shock, via axon severing, grey matter hemorrhage and necrosis, microglial activation, and upregulation of proinflammatory cytokines (IL-1β, IL-1α, TNFα, and IL-6) [8,9,10,11,12,13,14,15]. Traumatic demyelination of damaged axons occurs at the lesion site [16]. At the onset of injury, spinal cord swelling presides first, with gray matter and then white matter hemorrhaging secondary to vascular disruption [17,18]. While such findings may be evident pathologically, magnetic resonance abnormalities can be absent within the first two hours of injury, notwithstanding major structural disruption [19,20]. Given primary injury rarely fully transects the spinal cord, there is often the persistence of spared dysmyelinated or demyelinated axons traversing the injured substrate [8,9,21,22,23,24]. Animal models have demonstrated that preservation of even 5% of the initial axons can yield significant neurofunctional sustenance [8,9,25,26]. Such observations could account for the observed benefits from immediate decompression of the spinal cord during primary injury in humans [8,9,27,28].

Secondary injury temporally follows the primary phase and can be categorized as early acute (within 48 h of injury), secondary subacute (within 14 days), intermediate (within 6 months), and chronic (past 6 months) [8,9]. During the early acute period (2–48 h), cord inflammation, edema, and hemorrhage continue to progress, yet are compounded by free radical mediated injury, loss of ionic homeostasis, glutamate-induced excitotoxicity, permeability of the blood–brain barrier, inflammatory cell infiltration, demyelination, and cell death [8,29,30,31,32,33,34,35,36,37,38]. Demyelination and Wallerian degeneration progress in both a rostral and caudal manner to the lesion in this secondary phase [16]. After, the subacute phase (2–14 days) ensues, which is marked by a maximal phagocyte response and initiation of reactive astrocytosis (astroglial scar formation) [8,31,39]. In the intermediate phase (2 week to 6 months), the astrocytic scar continues to mature, yet simultaneously corticospinal (3 weeks to 3 months) and reticulospinal tract (3 to 6 months) axons begin sprouting, highlighting the regenerative potential at even 6 months post-injury [8,40]. Finally, from 6 months and beyond, the chronic phase initiates, where the SCI lesion continues scaring with development of cysts and syrinxes [8,9]. At roughly 1–2 years post-injury, the lesion stabilizes and potential for neurologic recovery ceases; however, with syrinx formation, delayed neurofunctional deterioration may occur in an estimated 30% of patients as the syrinx expands, inducing ascending paralysis, brainstem symptoms, or neuropathic pain [8,9,41,42].

Studies involving animal models have recognized the subacute phase as the critical period to promote functional recovery via neural transplantation—cells transplanted prior or after the subacute phase often fail to stimulate functional recovery [43,44]. The optimal milieu for transplantation likely arises secondary to the significant macrophage infiltration—which removes cell debris, including growth-inhibitory molecules—and astrocyte-mediated interventions (blood–brain barrier repair, edema resolution, ionic homeostasis reestablishment, and limitation of immune cell infiltration) [8,31,39,43,45].

In summary, while there may be potential for some neurofunctional recovery at 1–2 years post-injury, given the current understanding of SCI pathophysiology, earlier intervention (especially at the primary and sub-acute phases) likely imparts the greatest benefit for patients. Ideally, cellular treatments could coincide with the timing of surgery, which, when carried out within 24 h, improves outcomes and provide neuroprotection in the acute phase [28].

In terms of congenital SCI, myelomeningocele (MMC), the most severe form of spina bifida, is thought to derive from a “two-hit” hypothesis. The “first hit” is the failure of neural tube closure. The vertebral arches are spread wide, the dura is open and fused to the deep dermis of the skin, and the open pia mater is not covering neural tissue but rather connected to superficial dermis and epidermis [46,47]. The “second hit” is degeneration of the spinal cord in utero [48,49]. The developing spinal cord is not only exposed to physical trauma within the uterus but also the enzymatic activity of amniotic fluid [50]. Direct trauma can be visualized as focal hemorrhages and abrasions of the spinal cord at birth. In a study of MMC fetuses there were varying degrees of preserved neural tissue at the MMC defect [46]. This secondary injury is the rational for fetal repair for spina bifida [51].

In a retinoic acid induced rat model of MMC, fetal spinal cords exposed to amniotic fluid showed astrogliosis, neuroinflammation and reactive microgliosis [52]. Elevated glial fibrillary acidic protein (GFAP) can also be seen in amniotic fluid, signifying neural tissue degeneration [53]. Significant cord edema is also present in animal models of MMC, similar to what is seen in acquired SCI pathology [52,54]. Studying the mechanisms of secondary injury in both types of SCI could inform targeted therapy to inhibit inflammation and support neuronal and glial cell survival and recovery.

## 3. Properties of Placental Mesenchymal Stem/Stromal Cells

### 3.1. Isolation and Culture

Placental stem/stromal cells (PMSCs) are typically harvested through an explant culture method from donated placentas or chorionic villus sampling. Lankford et al. described methods to isolate, expand, and bank early gestational PMSCs for use as stem cell therapy [55,56]. In brief, the chorionic villus tissue is carefully dissected from placental tissues then washed in a sterile solution of phosphate-buffered saline (PBS) containing 100 U/mL penicillin and 100 g/mL streptomycin, minced into <1 mm^3^ fragments and then cultured and maintained in stem cell culture media. These methods are extremely feasible and can generate cell populations on the order of 10^9^ cells in less than four weeks. PMSCs show higher plasticity compared with MSCs derived from adult tissue sources [7]. In a comparison study to bone marrow, umbilical cord, and adipose-derived MSCs, PMSCs had the highest proliferative capacity and could withstand longer periods of culture [57]. Barlow et al. demonstrated similar results showing superior proliferation capabilities at different seeding densities compared with more commonly sourced bone marrow MSCs [58].

### 3.2. PMSC Characterization

PMSCs have demonstrated multipotency and can differentiate into osteogenic, adipogenic, and chondrogenic phenotypes [55]. There have been reports of PMSC differentiating into neurons, oligodendrocytes, and glial cells [59,60,61]. PMSCs have also been shown to be positive for well-established MSC surface markers CD105, CD90, CD73, CD44, and CD29 [56]. Transcription factors Sox10 and Sox17 and intracellular stem cell-related proteins Nestin, S100β, and neurofilament medium have been found in PMSCs [56]. Wang et al. cultured PMSCs in a collagen hydrogel and found they secreted a wide variety of growth factors in the first 24 h of culture. The highest were urokinase plasminogen activator (uPA), vascular endothelial growth factor (VEGF), TIMP-1, hepacyte growth factor (HGF), thrombospondin-1, monocyte chemoattractant protein 1 (MCP-1), interleukin (IL)-8, serpin E1, serpin F1, and macrophage migratory inhibitory factor, all of which play key roles in angiogenesis, chemotaxis, extracellular matrix remodeling, and innate immune responses [62].

Similar to bone marrow-derived MSCs (BM-MSCS), PMSCs also express higher levels of alpha 4 beta 1 (α4β1), also known as very late antigen (VLA)-4 which facilitates endothelium adherence and helps MSCs home to sites of injury [63]. VLA-4 expression on MSCs can interact with inflammatory cell subsets, including circulating leukocytes and modulate infiltration of cell subsets into inflamed tissues. Therefore, expression of VLA-4 by PMSCs can modulate migration through binding the endothelium of the blood–brain barrier (BBB) into the central nervous system (CNS) [1,64].

### 3.3. PMSC-Secretome—Free Proteins 

The efficacy of MSCs is understood to occur primarily through paracrine signaling effects rather than direct cell engraftment [1,65,66,67,68]. Cytokine arrays and single cell analysis have studied the secretome of PMSCs, which is key to understanding the differences between various MSCs. Early gestation PMSCs when compared with BM-MSCs secreted significantly higher levels of brain-derived neurotrophic factor (BDNF) and HGF [62]. BDNF has been shown to be an important regulator in neuronal differentiation [69]. BM-MSCs applied in stroke and SCI animal models correlated increased levels of BDNF with nerve growth factor and demonstrated improved motor function [70].

### 3.4. PMSC Secretome—Extracellular Vesicles (EVs)

The secretome of PMSCs also includes extracellular vesicles (EVs). EVs are membrane-bound nanovesicles containing proteins, DNA and RNA, and can be classified as apoptotic bodies, microvesicles, and exosomes [71]. EVs have the ability to reach sites of injury in the CNS by naturally crossing the BBB [72,73]. Nanosized exosomes could also more efficiently reach the target organ than larger MSCs [74]. Advantages of MSC-derived EVs over cellular therapy include safety, lack of tumorigenicity, and lower immunogenicity [73,74,75]. Evs also have the potential to be a readily available cell-free, off the shelf product as opposed to MSCs, which need to be thawed, cultured, and maintained before clinical applications.

Evs can be isolated through ultracentrifugation, precipitation, immunoaffinity size-exclusion chromatography, and ultrafiltration methods. Consistent isolation and purification at a large scale remains a limitation for all MSC-derived EVs [71]. Heterogenicity of EV preparations can make the mechanism of action studies challenging; however, this is an active area of research and new optimizations are constantly being developed. Standardization of intervention strategies will be key for translation applications using PMSCs or PMSC-EVs for the treatment of SCI.

## 4. Potential PMSC Therapeutic Mechanisms 

### 4.1. Neuroprotection

Proteomic analysis of PMSC EVs revealed the presence of several proteins and RNAs involved in neuronal survival. As discussed, free proteins such as growth factors BDNF and HGF have neuroprotective properties. Conditioned medium (CM) and CM supplemented with HGF from bone marrow MSCs was shown to promote neural stem cell differentiation and neurite outgrowth in a rat model of SCI. [76]. Additionally, human BM-MSCs genetically modified to overexpress BDNF-preserved cortical neurons, and promoted axon sprouting, resulting in functional recovery in a rat model of acute SCI [77]. Additionally, galectin-1, an evolutionarily conserved β-galactoside-binding lectin, has been shown to have neuroprotective and immunomodulatory functions, is also highly expressed on PMSCs. [78] While PMSCs have been shown to have increased expression of several mediators including BDNF, HGF, and galectin-1, leading to superior neuroprotective effects, these mediators have multifaceted functional properties and can also be involved in the immunomodulatory and pro-angiogenic properties of PMSCs.

**Figure 1:**
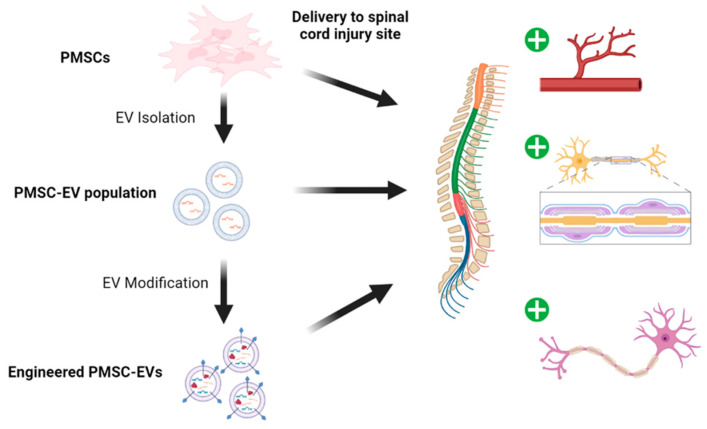
PMSCs, PMSC-EVs and engineered PMSC-EVs can each confer therapeutic effects in SCI by stimulating angiogenesis, remyelination, neuroprotection, and reducing inflammation.

MSC-derived EVs can cross the blood–brain barrier, making them a promising courier for CNS targeting (Figure 1). PMSC-EVs have potent neuroprotective properties and contain proteins and RNAs which protect neurons from apoptosis induced by staurosporine [78]. Indirect coculture of PMSCs, PMSC EVs with apoptotic SH-SY5Y cells, showed the ability of PMSCs and PMSC EVs alone to protect dying neurons. Subsequent, neuron analysis showed an increased number of branching points, circuitry length, and tube length [78].

### 4.2. Immunomodulation

The placenta, which helps maintain fetal tolerance, contains cells which display distinctive immunomodulatory properties [7]. Targeting the immune response in SCI has been studied with the testing of drugs such as minocycline and VX-210/BA-210 [15,79,80,81].

Following injury in the spinal cord, damage to resident oligodendroglia, axons, and neurons will occur due to mechanical injury, oxidative stress, and robust pro-inflammatory cytokines and chemokines. Modulation of pro-inflammatory responses including interleukin (IL)-1β, IL-6, IL-8, MCP-1 and TNFα have been shown to attenuate symptoms associated with SCI [82,83,84]. Neuroinflammation and microglia activation are seen in several neurodegenerative diseases and contribute to chronic neurodegeneration after SCI [85]. Macrophages are essential for reconstruction of repaired tissue but can also damage neurons and glia. Macrophages in the spinal cord arise from activated resident microglia and blood-borne monocytes, the latter of which are believed to be effectors of secondary injury [86]. Within microglia, two phenotypes—M1 and M2—have been identified and play unique roles in SCI pathology; the M1 phenotype is pro-inflammatory and the M2 phenotype, by contrast, is anti-inflammatory [81]. MSC-derived EVs have been shown to modulate macrophage and microglia polarization by modulating M1 pro-inflammatory responses and driving an M2 anti-inflammatory phenotype [87]. Prolonged M1 responses can have detrimental effects in the context of SCI, and M2 phenotypes can promote axonal regeneration [84,88,89]. 

Several studies have reported that BM MSC-EVs improve SCI outcome by reducing microglia activation of neurotoxic reactive astrocytes [90]. BM-MSC EVs may also modulate activation of astrocytes by reducing NF-κB signaling pathways and complement signaling activation [91]. Lee et al. demonstrated that PMSCs are more likely to have a stronger immunosuppressive function versus bone marrow- and adipose-derived MSCs due to increased expression of HLA-ABC and HLA g in PMSCs compared with others [92]. Similar results were seen by Chang et al. which showed decreased lymphocyte reactivity and increased numbers of regulatory T cells after mitogen or alloantigen stimulation [93]. PMSCs have also shown strong immunosuppressive effects in graft versus host disease animal models [94,95]. MSCs from several tissue sources have been shown to reduce lymphocyte proliferation and reduce pro-inflammatory mediators in vitro [94,96]. In a canine model of inflammatory brain disease, PMSCs reduced TNFα-induced inflammatory responses in vitro by attenuating pro-inflammatory cytokines and inhibiting leukocyte proliferation by inducing apoptosis [97]. Additionally, canine PMSCs were shown to be more robustly immunosuppressive compared with ASCs, which were found to inhibit leukocyte proliferation causing cell cycle arrest [97]. These findings demonstrate that while MSCs from differing tissue sources are immunosuppressive the mechanism of action differs, suggesting therapeutic intervention strategies may need to be optimized for different disease targets.

MSC-mediated immunosuppression has been suggested to occur in part by the induction of regulatory T cells. The unique properties of PMSCs have been illustrated in T cell responses to mitogen and allogeneic cell simulations [93,98]. One mechanism for these properties is the expression of programmed death ligand (PD-L)1 and FasL immunomodulation mediated by PMCSs [99]. Promotion of a regulatory T cell subset has been shown in the experimental autoimmune encephalitis (EAE) model to promote oligodendrocyte differentiation and re-myelination [100]. The findings from this study demonstrate modulating the microenvironment and immune response in SCI, which can lead to downstream effects that preserve and promote regeneration of neural cell subsets including myelination oligodendrocytes.

### 4.3. Myelin Regeneration

Myelin regeneration is an attractive therapeutic target for SCI in the interest of regaining functional motor outcomes. Following injury, remyelination occurs as oligodendrocyte precursor cells home to damaged axons, a process which can be modulated by resident immune cells including astrocytes and microglia [101]. Environmental cues will promote differentiation of oligodendrocyte precursor cells (OPCs) to become mature myelinating oligodendrocytes. Therefore, intervention strategies for CNS disorders including SCI target the promotion of remyelination by altering the injured microenvironment to promote OPC maturation or replacing damaged or destroyed myelinating oligodendrocyte populations [101]. MSC-EVs have been studied for their potential to promote myelin regeneration. In an EAE model of multiple sclerosis (MS), PMSC EVs increased re-myelination by the maturation of oligodendrocyte precursor cells to mature oligodendrocytes [102]. HGF has been shown to play a role in remyelination through promoting development of oligodendrocytes and neurons in models of EAE [103]. The mechanism by which PMSC and PMSC-EVs promote remyelination is multifaceted. The ability of PMSCs and PMSC-EVs to modulate inflammatory responses may create a microenvironment that allows for superior OPC homing and maturation thus resulting in re-myelination. Additionally, these cells and nanoparticles can also interact directly with oligodendrocyte populations to promote re-myelination. Many studies have demonstrated MSC and MSC-EV promotion of myelination of chronic MS; however, the mechanism of action is not fully elucidated in the literature [104,105]. Therefore, further studies are warranted and in the context of SCI and acute injury.

### 4.4. Angiogenesis

SCI causes vasculature disruption and compromise of the blood–spinal cord barrier. The following ischemia and inflammation can also cause further damage and neurological deficits. Given these vascular changes after SCI, understanding the regulation of angiogenesis is key, as angiogenesis facilitates post-SCI recovery and plays an important role in SCI repair [106,107]. MSCs secrete proangiogenic factors such as VEGF, HGF, and BDNF [108]. Hao et al. demonstrated that early gestational PMSCs had high-level secretion of VEGF, HGF, and BDNF, indicating that early gestational PMSCs possess strong angiogenic properties [109]. In addition, MSC-derived EVs have recently been used for therapeutic angiogenesis in ischemic disease [110]. Hao et al. also demonstrated that PMSC-EVs provided strong angiogenic properties via in vitro EC migration and ex vivo aortic ring sprouting assays [111].

## 5. PMSC and PMSC-EV Bioengineering

### 5.1. Delivery

Scaffolds and injectable hydrogels have been designed to enhance survival and support potential stem cell engraftment and differentiation of various cell types in injured tissue [2]. PMSCs have been seeded on collagen hydrogel and fibrin glue as well as nanofiber scaffolds made of synthetic polymers [56]. In contrast to injected cell therapies, bioscaffolds limit mass diffusion away into the vasculature, can be applied directly to injury sites, and allow for sustained release of therapeutic secretions. PMSCs have been shown to adhere well onto collagen and electrospun scaffolds, and in this 3D culture, can even differentiate into neuronal-like cells while attached to the scaffold [112]. This expands their potential and can be applied to areas of neuronal cell death, such as in SCI. 

The viability of PMSCs on an SIS-ECM scaffold has been validated by Lankford et al., with an optimal seeding density between 3 × 10^5^ and 5 × 10^5^ cells/cm^2^ [55]. MSCs seeded on SIS-ECM have also been shown to release more pro angiogenic factors, specifically VEGF and IL-8 [113]. MSC-derived EVs have also been shown to adhere well to both organic and synthetic bioscaffolds, and can still impart their therapeutic effects when implanted.

Hypoxic preconditioning can also be used to improve survival and engraftment of MSCs derived from different tissue. Hao et al. have demonstrated that hypoxic preconditioning significantly improved survival and angiogenic secretion of early gestational PMSCs [109,114,115].

### 5.2. Surface Modification

One limitation of EVs in treating CNS disorders is the issue of biodistribution after systemic administration. In some studies, EVs have been seen to accumulate in the spleen and liver and very few are detected in the CNS [116]. To address this limitation, ligand modification of EVs can improve longevity and cell- or tissue-specific targeting. It has been shown that EVs modified with a rabies virus glycoprotein (RVG) peptide cross the blood–brain barrier and deliver siRNA and miRNAs to injured brain tissue [117,118,119]. Another peptide, targeted axonal import (TAxI), can deliver protein cargo into spinal cord motor neurons by retrograde transport after intramuscular injection [120]. PMSC-EV plasma membranes can easily carry modified peptides and transmembrane proteins, and often carry “self” markers characteristic of their parent cell type [121]. Hao et al. developed an approach to utilize integrin α4β1, highly expressed on surfaces of PMSCs and PMSC-EVs, to bind ligands with various capabilities on to the PMSC-EV surface [111,122]. The capability of EVs to retain desired surface modification and cross the BBB make EVs ideal for the targeted delivery of neuroprotective therapy.

### 5.3. Cargo Loading

EVs naturally contain small RNAs including microRNAs (miRNAs), which have numerous signaling and regulatory roles in the CNS, such as immunomodulation and spinal cord regeneration. EV contents can easily be modified to contain miRNAs and siRNAs for site-specific delivery [123]. For example, miR-210-loaded MSC EVs promoted angiogenesis and improved the survival rate of rats in a model of cerebral ischemia [124]. miR-124 is known to play a role in neurogenesis and brain homeostasis. miR-124-loaded EVs delivered intranasally in mice attenuated the activation of inflammatory microglia [125]. PMSCs EVs have the potential to be loaded with miRNA, proteins, or pharmaceuticals to treat SCI.

## 6. Applications of PMSCs in SCI

### 6.1. Congenital SCI

PMSCs are the first stem/stromal cells to be studied in a clinical trial for the fetal treatment of congenital SCI. Preclinical work involved ovine and rat models of myelomeningocele. Fetal rats with MMC treated with PMSCs displayed less spinal cord compression and decreased apoptotic cell density in an ovine model; an MMC defect was surgically created in fetal sheep and then later repaired in utero [62,126,127]. In these studies, PMSCs seeded on an ECM were applied directly to the exposed spinal cord before repairing the open defect. Only lambs who received PMSC cell treatment were able to ambulate after birth. The density of large neurons was significantly greater in cell-treated lambs and correlated with motor score. There was no engraftment of GFP-labeled PMSCs in the spinal cords or tissues, suggesting a solely paracrine effect [62]. Although the mechanism is not fully elucidated, PMSCs in the fetal environment seem to augment healing and tissue regeneration.

### 6.2. Acquired SCI

A few groups have begun to investigate PMSCs in SCI. In a canine transection SCI model, PMSCs from discarded term placentas seeded on a linear ordered collagen scaffold promoted neuronal regeneration and functional motor recovery [128]. Histological analysis revealed decreased CSPG deposits, increased axon regeneration and remyelination, and increased ascending and descending sensory nerve fibers [128]. Zhou et al. demonstrated PMSC-EVs injected into a rat model of transectional SCI, PMSC-EVs alleviated neurogenic bladder function, promoted functional recovery by activating endogenous progenitor cell markers, stimulated neural stem cell proliferation, and were selectively taken up by neural stem cells [129]. Li et al. described an innovative implantation of PMSC-EVs that were immobilized in a peptide-modified adhesive hydrogel and delivered directly into injured nerve tissue in a rat long-span spinal cord transection model; the implantation demonstrated significant nerve recovery and urinary tissue preservation by effectively mitigating inflammation and oxidation [130].

In a mouse spinal cord contusion model, PMSC-EV application was associated with improved angiogenesis and tube formation via human umbilical cord vein epithelial cell (HUVEC) migration, and hindlimb sensory and locomotor responses [131]. The proangiogenic properties of PMSCs are beneficial as SCI causes local vascular damage, which can lead to ischemia and limit proper healing.

PMSCs and PMSC-EVs have also been applied to treat SCI caused by other reasons, such as multiple sclerosis [128]. Several other preclinical studies involving MSC-EVs have been conducted in recent years, proposing that MSC-EVs can induce angiogenesis, axon formation, regulate inflammation and the immune response, inhibit apoptosis, and maintain integrity of the blood–spinal cord barrier [132].

**Figure 2:**
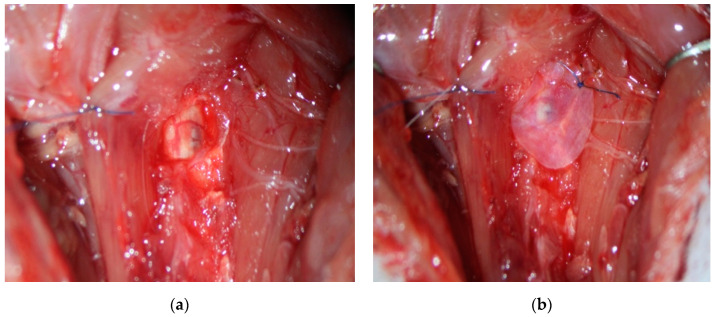
Surgical implantation of PMSCs seeded on an extracellular matrix scaffold (SIS-ECM) to a rat C5 spinal cord hemicontusion model. (**a**) Contused spinal cord visible after C5 hemilaminectomy and impact in an adult rat. (**b**) Placement of PMSCs seeded on SIS-ECM scaffold directly placed onto spinal cord after dura was opened.

In a preliminary animal study, we applied our PMSCs and PMSC-EVs seeded on an extracellular matrix scaffold (SIS-ECM) to a rat C5 spinal cord hemicontusion model. As shown in Figure 2, C5 spinal cord hemicontusion was performed after hemilaminectomy (Figure 2a). PMSCs seeded on SIS-ECM were then directly placed onto the injured spinal cord after dura was opened (Figure 2b). The preliminary study showed that PMSCs seeded on SIS-ECM can be surgically applied to the local SCI site. Similar to the clinical trial for fetal repair of spina bifida, PMSCs seeded on a SIS-ECM scaffold can be placed directly on the injured spinal cord (Figure 2). Translationally, the SIS-ECM scaffold can serve as a dural graft, if a duroplasty is performed during the decompression of the spinal cord after SCI.

## 7. MSCs and PMSCs in Clinical Trials

There are currently several active clinical trials utilizing MSCs in SCI; however, none use PMSCs as a source [133,134,135] (Table 1). Cells can be delivered intravenously, intrathecally, or intramedullary. The primary outcomes for most trials are improvement in motor function typically measured by the American Spinal Injury Association Impairment (ASIA) Scale (AIS) Score.

These current SCI clinical trials utilize MSCs derived from bone marrow, adipose, and umbilical cord. Umbilical cord MSCs (UC-MSCs) also derived from perinatal tissue and fetal origin are more like PMSCs than bone marrow or adipose cells [136]. UC-MSCs have been demonstrated to decrease astrogliosis in SCI, downregulate T-cell-mediated inflammation, and survive long after transplantation [137,138,139]. Adipose-derived MSCs have been associated with increased angiogenesis, axonal regeneration, and extracellular matrix formation [140,141]. PMSCs with their neuroprotective, angiogenic, and antioxidant are ready to be added to the milieu of MSC sources for spinal cord injury. Future research may seek to compare MSC sources to elucidate their individual effects and ability to treat specific diseases.

Cellular therapy for in utero repair of myelomeningocele – The CuRe Trial, is the first clinical trial utilizing MSCs for congenital SCI [142]. PMSCs used in the CuRE Trial and are also being investigated in other diseases. PMSCs due to their immunomodulatory effects have been effective in treating steroid refractory acute graft-versus-host disease; Ringden et al. showed a remarkable 73% survival rates in patients versus 6% in retrospective controls in a 1-year follow-up [143]. PMSCs have also been used to treat epidermolysis bullosa, a severe blistering skin disease that causes multiple cutaneous wounds from birth [144]. There are currently trials utilizing PMSCs in treating diabetic foot ulcer, COVID-19 pneumonia, and osteoarthritis [145,146,147].

## 8. Conclusions

The feasibility and safety of PMSCs and PMSC-EVs should encourage their use in more preclinical and clinical trials for SCI. Additionally, PMSCs and PMSC-EVs have shown promise in site-specific targeting and could be optimized for more precise targeting and specialized cargo delivery or downstream regenerative cell signaling cascades. PMSCs are easily isolated and can lend their immunomodulatory, antioxidant, angiogenic, and neuroprotective capabilities to SCI treatment. In summary, PMSCs and PMSC-EVs have unique functional and therapeutic properties making them ideal targets for developing treatments for SCI.

## Figures and Tables

**Table 1:** Active clinical trials utilizing mesenchymal stem cells for spinal cord injury.

Trial Identifier	Name	Phase	Key Inclusion Criteria	Therapy
NCT03505034	Intrathecal Transplantation of UC-MSC in Patients with Late Stage of Chronic Spinal Cord Injury	2	Age 18–65 ASIA Scale A–D Injury more than 12 months	Intrathecal Transplantation of UC-MSCs, 10^6^ cells/kg, once a month for 4 months
NCT03521336	Intrathecal Transplantation of UC-MSC in Patients with Sub-Acute Spinal Cord Injury	2	Age 18–65 ASIA Scale A–D Injury 2 weeks to 2 months	Intrathecal Transplantation of UC-MSCs, 10^6^ cells/kg, once a month for 4 months
NCT03521323	Intrathecal Transplantation of UC-MSC in Patients with Early Stage of Chronic Spinal Cord Injury	2	Age 18–65 ASIA Scale A–D Injury 2 months to 12 months	Intrathecal Transplantation of UC-MSCs 10^6^ cells/kg, once a month for 4 months
NCT02574585	Autologous Mesenchymal Stem Cells Transplantation in Thoracolumbar Chronic and Complete Spinal Cord Injury Spinal Cord Injury	2	Age 18–65 ASIA Scale A Injury T1-L2 with at least 12 months	Two percutaneous injections of MSCs, with a 3-month interval between the injections
NCT05018793	Safety of Cultured Autologous Adult Adipose-Derived Mesenchymal Stem Cell Intrathecal Injection for SCI	1	Diagnosis of Spinal Cord Injury	Single intrathecal injection of 100 million cells
NCT02688049	NeuroRegen Scaffold™ Combined with Stem Cells for Chronic Spinal Cord Injury Repair	1, 2	Age 18–65 ASIA Scale A Injury C5-T12	Patients with chronic SCI (ASIA grade A) will receive NeuroRegen Scaffold with 10 million MSCs transplantation after localized scars cleared
NCT04520373	Autologous Adipose-Derived Mesenchymal Stem Cells for Spinal Cord Injury Patients	2	Age >18 ASIA Scale A, B	Patients will receive a single dose of autologous, adipose-derived MSCs one time
NCT03308565	Adipose Stem Cells for Traumatic Spinal Cord Injury (CELLTOP)	1	Age >18 ASIA Scale A, B Injury 2 weeks to 1 year	Patients will receive a single dose of 100 million autologous, adipose derived MSCs
NCT02917291	Safety and Preliminary Efficacy of FAB117-HC in Patients with Acute Traumatic Spinal Cord Injury	1, 2	Age 16–70 Phase 1—ASIA A, Injury 72–120 h prior Phase 2—ASIA A, B, Injury up to 96 h prior	Intramedullary administration of FAB117-HC (Adipose Derived Adult MSCs Expanded and Pulsed with H_2_O_2_)
NCT04213131	Efficacy and Safety of hUC-MSCs and hUCB-MSCs in the Treatment of Chronic Spinal Cord Injury	n/a	Age 20–65 ASIA A-D with no change Injury over 1 year	Intravenous, lumbar, and local administration of hUC-MSCs/hUCB-MSCs

## Data Availability

No new data were created or analyzed in this study. Data sharing is not applicable to this article.
